# Treatment Adherence and Quality of Life Among Elderly Patients With Diabetes Mellitus Registered in the Community

**DOI:** 10.7759/cureus.58986

**Published:** 2024-04-25

**Authors:** Eirini Vafopoulou, Nikolaos Christodoulou, Ioanna V Papathanasiou

**Affiliations:** 1 Management of Health Units, Hellenic Open University, Patra, GRC; 2 Department of Psychiatry, Faculty of Medicine, University of Thessaly, Larissa, GRC; 3 Community Nursing Lab, Faculty of Nursing, University of Thessaly, Larissa, GRC

**Keywords:** community, medication adherence, elderly, quality of life, diabetes mellitus

## Abstract

Background

This study investigates the association between medication adherence and health-related quality of life among adults with type 2 diabetes mellitus at the Health Center of Tyrnavos community level.

Materials and methods

This cross-sectional study involved 125 patients with type 2 diabetes mellitus, aged 60 years and older, who were visiting community healthcare facilities. The research was conducted with a structured questionnaire that included 34 questions related to socio-demographic data, self-reported compliance, and stress. The DQOL-BCI (Diabetes Quality of Life - Brief Clinical Inventory) scale was used to measure health-related quality of life.

Results

A total of 125 patients with a mean (SD) age of 69.2 (8.1) years were included in the study (64 women and 61 men). Based on the results of the descriptive analysis, 88.0% reported high medication adherence. However, 66% of the participants reported having high anxiety levels, with 33.6% having difficulty controlling their anxiety. Quality of life was negatively correlated with lower medication adherence (*P* < 0.05).

Conclusions

Older age and low medication adherence are associated with lower quality of life among diabetic patients. Interventions to improve the quality of life in elderly diabetic patients should consider the effect of age and medication adherence.

## Introduction

Type 2 diabetes mellitus (DM) is a chronic progressive disease with high prevalence, and it poses a significant challenge to healthcare systems worldwide [1-2]. Diabetes in Greece follows the prevalence patterns of other developed countries, and the estimated prevalence of type 2 diabetes in the population is 4.11%, based on 400,000 cases [3].

DM is associated with an increased prevalence and incidence of geriatric syndromes among elderly individuals, including functional disabilities, depression, falls, urinary incontinence, malnutrition, and cognitive impairment. Geriatric syndromes not only lead to frailty, loss of independence, and low quality of life (QOL), but they also represent a major obstacle in the treatment and care of people with diabetes [4]. The number of deaths from DM in the age group 60-99 accounted for 60% of the total number of deaths among elderly patients, or 3.2 million individuals, in 2017 [5].

Diminished perception of hyperglycemia symptoms, a consequence of normal changes associated with ageing, and the expected worsening of geriatric syndromes also play a part in reduced treatment compliance among elderly diabetic patients [6-8]. Elderly patients with chronic health conditions face many challenges, which are intensified in the context of social exclusion and diminished family support. Polypharmacy and comorbidity of chronic diseases intensify noncompliance with medication and exacerbate poor QOL [9-11]. Patients with DM also need to follow instructions on an appropriate diet and maintain a stable body weight, which, in combination with older age and the coexistence of a mental condition, negatively affects the QOL [12].

DM may also have adverse effects on both the personality and the emotional status of an individual. Negative factors for QOL for diabetic patients include low income, low capacity for self-care, and complications of the disease, especially when hospitalization is necessary [13-15].

In recent studies on health literacy, high compliance with pharmaceutical treatment is related to better QOL for a DM patient. Alfian et al. [16] and Zioga et al. [17] reported similar results from their studies with 91 patients (most over 60 years old) and 108 patients (average age of 67 years), respectively; both studies showed a positive correlation between treatment maintenance and the QOL in DM patients. The best QOL was associated with high compliance with antidiabetic treatment and appeared to be positively affected by female sex and lower body mass index [18]. Another study with 300 patients with DM showed that medication compliance was an independent positive predictive factor for the patients’ QOL [19].

The relationship between medication compliance and the QOL among patients with DM is of utmost importance in creating appropriate programs and planning interventions that will benefit and educate patients [20]. This study aimed to research pharmaceutical treatment and QOL of elderly patients with DM in the community and a possible association between them.

## Materials and methods

Sample and settings

A cross-sectional study was conducted by collecting data at the outpatient clinics of the Health Center of Tyrnavos and the outpatient rural clinics within its territory from January 2021 to March 2021. The research was conducted with the participants’ free and voluntary consent, using a specially adapted questionnaire for elderly patients with DM.

The study population consisted of a convenience sample of 125 patients. The inclusion criteria were the following: consent to participate in the study, 60 or more years of age, ability to communicate in the Greek language, and an official diagnosis of DM.

Ethical considerations

First, it was necessary to secure the consent of the participants in the research process before they signed the consent form. Before completing the questionnaires, patients were informed that their participation was voluntary and anonymous. They were also informed that the collected data would be used exclusively for the study and that they could stop answering the questionnaires whenever they wished. In addition, for the questionnaire, DQOL-BCI (Diabetes Quality of Life - Brief Clinical Inventory) was used with permission from the developers [21]. The study protocol was approved by the 5th Regional Health Authority of Thessaly and Sterea in Greece (IRB approval No:46420/11.01.2021)

Data collection

Data were collected with a structured questionnaire, which included 34 questions related to the patient's demographic data, the chronicity of the disease, and adherence to treatment based on self-report, concerning the frequency of skipping the treatment and its possible interruption. The questionnaire also included two questions about anxiety. The validated Greek version of the questionnaire DQOL-BCI tool was used to measure satisfaction with different aspects of life among patients with DM [21]. The DQOL-BCI 15-item questionnaire was demonstrated to be reliable and valid by Rekleiti et al. [21]. It focuses on two parts of the patient’s life, with eight questions about factors with a positive effect on DM disease: “How satisfied are you…” with various parameters about treatment and QOL (such as diabetic treatment, time of diabetes regularization, time of diabetes definition, time for physical exercise, time for medical checkup, level of knowledge about diabetes, love life, the degree of distress caused by family members). The answers are ranked on a Likert scale from 1 (worst case, very dissatisfied) to 5 (best case, very satisfied). The second part of the DQOL-BCI includes seven questions beginning with “How often …” that refer to the negative effects of DM treatment, with a selection of multiple-choice answers ranked on a Likert scale from 1 (always, best case) to 5 (never, worst case). Through reverse rating of the answers to the questions on negative effects, a higher value reflects less negative effects and a higher QOL level. The questionnaire was translated and validated in Greek by Rekleiti et al. [21], and Cronbach’s alpha = 0.95 was used to test for validity and reliability [21].

## Results

Questionnaires were completed by 125 participants who met the study criteria. Quantitative variables are reported as mean values and standard deviations (SDs) and as medians and interquartile ranges. Absolute (N) and relative (%) frequencies are used to describe the qualitative variables. The parametric analysis of variance (ANOVA) or the nonparametric Kruskal-Wallis criterion was used to compare quantitative variables between more than two groups. Descriptive statistical analyses were applied to the results (e.g., sex, age, education, family status). The materiality level was conducted for the DQOL-BCI tool. The significance levels are bilateral, and the statistical significance was set at 0.05. The SPSS Statistics for Windows (Version 22.0, IBM Corp., Armonk, USA) was used for the analysis. The study population included 125 patients, aged 60 years and older, whose demographic characteristics are presented in Table 1.

**Table 1:** Demographic Characteristics of the Participants (N = 125)

Characteristics		n	%
Sex	Male	61	48.8
	Female	64	51.2
Age, years	60-65	47	37.6
	65-69	25	20.0
	70-74	20	16.0
	75-79	13	10.4
	80-84	12	9.6
	85-89	8	6.4
Family status	Married	96	76.8
	Unmarried	3	2.4
	Divorced	2	1.6
	Widowed	24	19.2
Place of residence	City	35	28.0
	Small city (>2000 citizens)	23	18.4
	Village	67	53.6
Residential status	Alone	20	16.0
	Not alone	105	84.0
Education status	Uneducated	5	4.0
	Primary education	65	52.0
	Junior high school	12	9.6
	High school	19	15.2
	University	17	13.6
	MSc or PhD	7	5.6
Profession	Privately employed	3	2.4
	Publicly employed	12	9.6
	Self employed	10	8.0
	Ηousehold	26	20.8
	Unemployed	3	2.4
	Retired	64	51.2
	Manual occupation (farmer, other worker)	7	5.6

**Figure 1:**
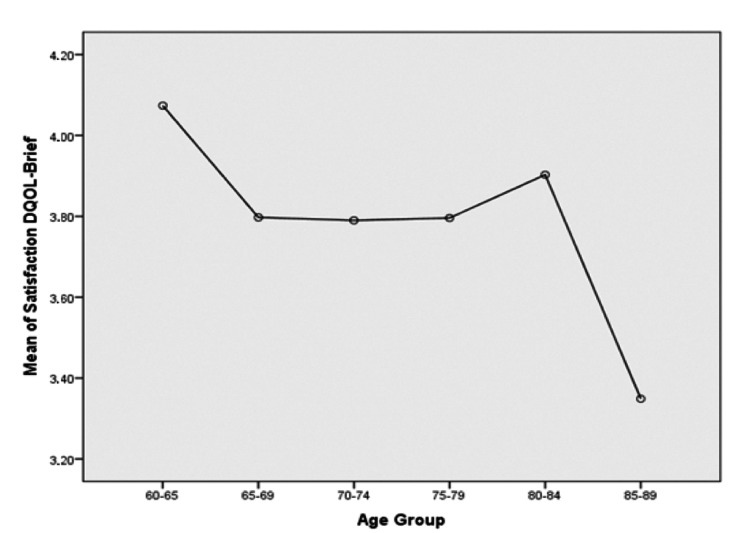
Average Satisfaction Diabetes Quality of Life Brief Clinical Inventory (DQOL-BCI) per Patients’ Age Group

Among the 125 participants included in the study, 51% were women and 62% were ≥65 years old. Sixty-seven (54%) of the participants lived in villages, and 70 individuals (56%) were primary school graduates. The parametric analysis of variance (ANOVA) in the current study showed that the place of residence, and educational status were correlated with lower compliance, and in addition, the older age was correlated with a lower impact on the patient’s QOL (Figure 1). 

The participants’ compliance with pharmaceutical treatment is presented in Table 2. They were asked whether they ever skipped taking their medication for DM, and if yes, how often. Among 125 participants, 110 (88.0%) reported that they had never omitted taking their medication. From the remaining 15 participants (12.0%), nine skipped medication doses once a month or less, while six patients skipped doses many times (more than once) in a month.

**Table 2:** Medication Noncompliance

Frequency of noncompliance	n	%
Never	110	88.0
Once a month or less	9	7.2
Many times	6	4.8
Total	125	100.0

The 125 participants were also asked whether they had ever skipped their treatment, and 118 (94.4%) reported no interruption of their suggested treatment. However, the cross-tabulation of interruption of treatment and frequency of omitting it in Table 3 presents a discrepancy between answers.

**Table 3:** Interruption of Treatment and Frequency of Omitting It

Frequency of omitting the treatment	Interrupted treatment		Total
	Yes	No	
Never	2	108	110
Once a month or less	2	7	9
Many times	3	3	6
Total	7	118	125

More specifically, 50% of the patients (three out of six) who missed their medication several times a month stopped the treatment at some point, while only 22% (two out of nine) who missed their medication once a month or less often stopped their treatment at some point. In contrast, among those who did not omit medication, 98% (108 out of 110) have never stopped their treatment.

The parametric analysis of variance (ANOVA) was used to compare quantitative variables between more than two groups. The compliance with the treatment was not related to age (P = 0.723), sex (P = 0.084), family situation (P = 0.852), level of education (P = 0.549), smoking habits (P = 0.242), weight (P = 0.246), height (P = 0.343), years of morbidity (P = 0.115), or type of medication (P = 0.983). On the contrary, compliance with medications was related to place of residence (P = 0.048), with participants living in the city showing greater compliance with medication than those in towns or villages. Compliance was also related to the profession (P = 0.048), with manual workers displaying less compliance than other professional groups.

Descriptive statistics

The participants’ responses to questions connected with the descriptive statistics from the DQOL-BCI were rated, as shown in Table 4.

**Table 4:** Responses to the Evaluation Criterion in the Diabetes Quality of Life Brief Clinical Inventory (N=125 for all criteria).

Question	Evaluation Criterion	Score				
		1	2	3	4	5
1	How satisfied are you with your diabetes treatment?	3	4	9	43	66
2	How satisfied are you with the time it takes for your diabetes to be controlled?	4	7	9	55	50
3	How often do you catch yourself eating something you shouldn’t, instead of telling others you have diabetes?	11	31	43	26	14
4	How often do you worry about whether you will be able to go to work?	3	8	11	20	83
5	How satisfied are you with the time it takes to test your blood sugar?	3	8	11	49	54
6	How satisfied are you with the time you spend on physical activity?	39	17	8	36	25
7	How often do you sleep poorly at night because of diabetes?	4	17	25	21	58
8	How satisfied are you with your love life?	39	23	24	23	16
9	How often do you feel that diabetes limits your career?	1	7	12	23	82
10	How often are you in pain because of diabetes treatment?	-	5	14	17	89
11	How satisfied are you with the extent to which your family has been burdened by your diabetes?	7	24	23	19	52
12	How often do you feel physically ill?	2	16	34	24	49
13	How often do you worry about passing out?	3	11	19	27	65
14	How satisfied are you with the time you have for medical control of your diabetes?	8	8	13	49	47
15	How satisfied are you with your level of knowledge about your diabetes?	4	13	18	54	36

The descriptive statistic elements (minimum value, average value, median value, maximum value, SD) for 15 assessment questions that related to the criteria of the DQOL-BCI questionnaire are included in Table 5. The minimum value is 1 and the maximum value is 5 for all criteria shown in Table 5. Further, the higher the median of a criterion, the higher the assessment from the participants. Higher median values imply either higher satisfaction or a lower frequency of negative effects from DM or the treatment for DM.

**Table 5:** Descriptive Data for Evaluation Criteria in the Diabetes Quality of Life Brief Clinical Inventory

Question	Evaluation Criterion	Average value	Median value	SD
1	How satisfied are you with your diabetes treatment?	4.32	5.00	0.92
2	How satisfied are you with the time it takes for your diabetes to be controlled?	4.12	4.00	0.98
3	How often do you catch yourself eating something you shouldn’t, instead of telling others you have diabetes?	3.00	3.00	1.12
4	How often do you worry about whether you will be able to go to work?	4.37	5.00	1.04
5	How satisfied are you with the time it takes to test your blood sugar?	4.14	4.00	0.98
6	How satisfied are you with the time you spend on physical activity?	2.92	3.00	1.57
7	How often do you sleep poorly at night because of diabetes?	3.89	4.00	1.22
8	How satisfied are you with your love life?	2.63	3.00	1.41
9	How often do you feel that diabetes limits your career?	4.42	5.00	0.93
10	How often are you in pain because of diabetes treatment?	4.52	5.00	0.84
11	How satisfied are you with the extent to which your family has been burdened by your diabetes?	3.68	4.00	1.33
12	How often do you feel physically ill?	3.81	4.00	1.13
13	How often do you worry about passing out?	4.12	5.00	1.11
14	How satisfied are you with the time you have for medical control of your diabetes?	3.95	4.00	1.14
15	How satisfied are you with your level of knowledge about your diabetes?	3.84	4.00	1.05

The participants appeared to have high satisfaction with diabetes treatment, time to medical control of DM, level of knowledge for DM, and burden on their family from the disease. The average satisfaction was close to 4 (from 4.32 to 3.68) and thus greater than 3, which is the average value of the Likert scale (i.e., neutrality). In contrast, the satisfaction with the time they devote to physical exercise was 2.93, which was slightly negative. The majority of the participants expressed dissatisfaction with their love life, with an average satisfaction of 2.63.

The most frequent negative behavior, with a mean value of 3.01, was related to food and eating something not allowed in the diet instead of telling others that they have DM. In contrast, the participants with less frequent, worry[JRB1] about being able to go to work, about feeling that diabetes limits their career, or about feeling pain from treatment for diabetes.

In analyses in which the general demographics were controlled for, the age of participants showed a statistically significant association with overall satisfaction. In contrast, no statistically important associations were found for average satisfaction with sex, family status, place of residence, residential status, level of education, and professional capacity.

Average satisfaction on DQOL-BCI and age

In parametric tests and specifically in a one-way ANOVA, there was a statistically important divergence of average satisfaction on DQOL with age (P = 0.021). In particular, older age appeared to have a negative association with DQOL.

Although the average satisfaction from the DQOL-BCI was not correlated with the level of education, it is important to emphasize that the answers to this particular question that measures satisfaction related to the level of knowledge about diabetes were correlated with the level of education. The satisfaction with the level of knowledge concerning DM appeared to be associated with the level of education.

There was a statistically significant divergence of satisfaction, about the level of education (*P* = 0.025). In particular, the higher the level of education of the patient, the higher the level of knowledge on the part of the patient concerning DM (Table 6).

**Table 6:** Association Between Satisfaction With the Level of Knowledge Concerning Diabetes Mellitus and the Level of Education

Education	n	Mean	SD	Confidence interval for mean (lower-upper bound)	Minimum	Maximum
Uneducated	5	3.40	0.54	2.71-4.08	3.00	4.00
Primary education	65	3.55	1.18	3.25-3.84	1.00	5.00
High school	12	4.08	0.79	3.57-4.58	2.00	5.00
High school	19	4.15	0.95	3.69-4.61	2.00	5.00
University	17	4.35	0.60	4.04-4.66	3.00	5.00
MSc or PhD	7	4.28	0.75	3.58-4.98	3.00	5.00
Total	125	3.84	1.05	3.65-4.02	1.00	5.00

Average satisfaction on DQOL-BCI and frequency of medication omission

The correlation of average satisfaction in the DQOL-BCI with omission of medication was also analyzed in Table 7. Because data did not follow normal distribution on the three levels of the independent variable “frequency of treatment omission,” nonparametric tests were used (Kruskal-Wallis Test). These tests revealed a statistically significant divergence of average satisfaction with the frequency of omission of pharmaceutical treatment.

**Table 7:** Correlation of Average Satisfaction in Diabetes Quality of Life Brief Clinical Inventory With Omission of Medication

Frequency of medication omission	n	Mean Rank
Many times a month	6	40.17
Once a month or more seldom	9	37.44
Never	110	66.34
Total	125	

The average satisfaction of patients concerning the frequency of omission of treatment is shown in Table 8. The patients who tended to omit the treatment appeared to have lower satisfaction, while those who never omitted treatment expressed higher satisfaction.

**Table 8:** Average Satisfaction of Patient in Relation to Frequency of Omission of Treatment

Frequency	N	Mean	SD	Confidence interval for mean (lower bound- upper bound)	Minimum	Maximum
Several times a month	6	3.4333	0.67032	2.7299-4.1368	2.33	4.13
Once a month or less	9	3.4524	0.52532	3.0486- 3.8562	2.60	4.20
Never	110	3.9409	0.67429	3.8135-4.0683	2.07	7.07
Total	125	3.8814	0.67956	3.7611-4.0017	2.07	7.07

**Figure 2:**
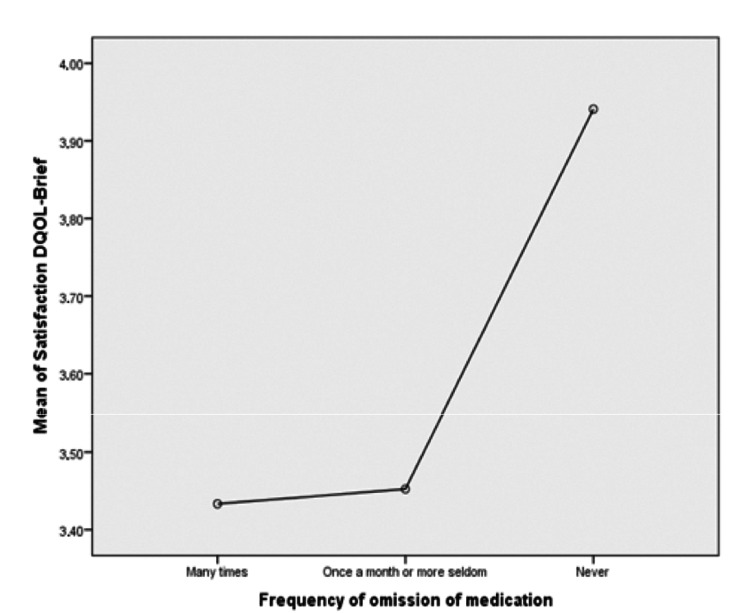
Mean Satisfaction Diabetes Quality of Life Brief Clinical Inventory and Frequency of Omission of Treatment

These results are presented in Figure 2.

## Discussion

This study aimed to investigate the association between medication adherence and health-related QOL among elderly people with DM. The compliance with taking prescribed medication was high (88%), which accords with previous studies [22]. The increased knowledge of patients regarding DM, which may be due to good communication between patients and doctors, could be responsible for the positive result [23]. The current study also showed that compliance with treatment was correlated with the place of residence, with participants who lived in cities having higher compliance than those residing in towns or villages [24]. Additionally, maintaining treatment appeared to be related to occupation, as manual workers were less likely to comply with treatment than participants in the other professional groups [25].

DM affects all aspects of QOL, as shown in several previous studies. In the current study, the greatest negative effects were detected in physical exercise, food intake, and love life, data that confirm results from other studies [26,27]. The average satisfaction on the QOL scale was close to 4 (higher than 3, the median value) on the DQOL-BCI, which accorded with other Greek studies, in which participants had high QOL [13]. In contrast, DM type 2 had a lower impact on patients’ QOL in some other studies [27,28].

However, in this study, elderly people appeared to show significant deterioration in QOL, as shown in other studies [26,27]. In a study involving 2,800 people that was conducted to study further DM in people with diabetes, old age was confirmed to be an aggravating factor for the QOL level [29]. Similar results were found by Donald et al. [13], who reported that older age, in combination with increased body weight and mental disease, had a negative effect on QOL. A Greek study that analyzed QOL in DM in the general population found a correlation of age with QOL standards, with results being similar to the current study showing old age as having a negative effect on QOL [13]. The present study focuses on elderly individuals, in whom further aging is expected to lead to low QOL [30].

The present study confirms research data that relate to compliance with medication and QOL in DM. The statistical analysis of various characteristics such as age, place of residence, level of education, and professional capacity, furthermore the individual questionnaires, reveal components with a positive or negative effect on health care and treatment of patients with DM.

There are also limitations to the research. The study population consisted of a convenience sample of patients residing in the area of a healthcare center and the regional medical centers under its control. Thus, the total sample size is small and might not be representative of the whole population. The results relied on the respondents’ self-reported data regarding their medication adherence and may have been subject to recall bias. In addition, the lack of data to confirm participants’ adherence to treatment (such as HbA1C results) may weaken conclusions. In addition, comorbid conditions were not included in the study since there were no questions related to other illnesses on the questionnaire. There may also have been subjective feelings on the part of the participants concerning the self-evaluation of their biochemical blood tests. Moreover, the attendance of the patients at clinics was limited because of the COVID-19 pandemic and the collection of questionnaires was quite difficult. Results may have been influenced by the pandemic and the extended confinement associated with it.

## Conclusions

The aging of the population and the increase in DM prevalence, in combination with noncompliance with medication among elderly patients, present a challenge for healthcare systems to improve geriatric interventions. In addition, the development of education individualized for each patient to address treatment challenges could increase patient satisfaction, leading to better compliance on medications.

The research on DM, as well as research on the compliance with medications and the QOL of diabetic patients, the recognition of the importance of demographic characteristics, and the need for decision-making in accordance with age highlights the importance of promoting health in the context of providing quality care to improve the QOL. The relation between compliance with diabetic treatment and QOL for DM patients constitutes a subject for future investigation aimed at developing appropriate programs and interventions for patient education.
